# “I Can’t Just Pull a Woman Out of a Hat”: A Mixed-Methods Study on Journalistic Drivers of Women’s Representation in Political News

**DOI:** 10.1177/10776990211073454

**Published:** 2022-03-03

**Authors:** Andreas A. Riedl, Tobias Rohrbach, Christina Krakovsky

**Affiliations:** 1 Austrian Academy of Sciences, Vienna, Austria; 2 University of Fribourg, Switzerland; 3 University of Amsterdam, The Netherlands

**Keywords:** gender representation, gender diversity, political journalism, mixed methods, journalistic roles

## Abstract

While the persisting issue of women’s underrepresentation in political news partly arises from biases in the social reality, journalism plays a crucial role in mediating these biases. This study proposes a multilayered framework of gendered influences in journalistic news production to understand how journalistic factors exacerbate or mitigate women’s media representation. Drawing from a mixed-methods design (content analysis, survey, interviews), journalists’ own gender emerges as the strongest predictor of gendered representations. Women’s underrepresentation is also influenced by professional roles but not by organizations’ gender guidelines. We explore how journalists perceive these influences and discuss conceptual and practical implications.

Women’s under- and misrepresentation in political news coverage has been problematized as a substantial breach of normative media diversity requirements (Napoli, 1999) and as a democratic deficit (Melischek & Seethaler, 2017). In societal institutions and in media coverage, “[d]iversity, as a pluralistic political aim, is a question of representation” (Sjøvaag & Pedersen, 2018, p. 215). A lack of women’s representation not only entails fewer role models that (young) women can identify with but also contributes to the cultivation of the political sphere as a “boyzone” (Ross, 2017, p. 17). Gerbner and Gross (1976) famously described the practice of media-driven marginalization as the “symbolic annihilation of women,” which acts as a mechanism to maintain a hegemonic status quo.

To gain a systematic understanding of influences on women’s representation in political news coverage, we combine literature on the gendered mediation of politics with research on journalistic gatekeeping to conceptualize gender representations as part of the larger social process of journalistic mediation. We theorize that gender influences in journalistic news production act as gatekeepers in the transformation of a political and social reality into a mediated reality. This broader conceptualization puts the understudied role of gender in the process of journalistic mediation on center stage; not only the media coverage itself is gendered but the journalistic mediation processes that precede and produce it. This conceptually widens past approaches, which have studied gender representations as differences in the amount and content of political media coverage between women and men, mostly in their role as politicians (cf. Hayes & Lawless, 2015; Kittilson & Fridkin, 2008; Meeks, 2012). Moreover, while past research has focused on more distant contextual drivers of gender representations rooted in political and social reality (e.g., electoral systems and gender equality norms), we follow current calls to explore the conceptually more proximate contextual influences in journalistic news production (Rohrbach et al., 2020; Sjøvaag & Pedersen, 2018).

The goal of this study is thus to systematize potentially relevant factors within journalistic news production, test their influence, and explore their negotiation within the news production process to gain a more holistic understanding of why women are (under)represented in journalistic news. Our empirical approach consists of a mixed-methods design combining (a) a manual quantitative content analysis of political news coverage in Austria, (b) a quantitative survey of the journalists authoring the analyzed news items, and (c) a series of qualitative reconstructive interviews with a subsample of the surveyed journalists.

## Understanding Women’s Representation: Gatekeeping in the Gendered Mediation Process

As a basic conceptual framework, the process of gendered mediation of politics can be broken down into three subparts (Gidengil & Everitt, 1999, 2003; Trimble et al., 2021): (a) the political and social reality, which represents the structural status quo of the world “out there”; (b) the mediation process involving the various selection and transformation subprocesses of journalistic news production; and (c) the mediated reality in the form of news coverage. The mediated (mis)representation of women then feeds back into the construction of the political and social reality. Existing research has concentrated on understanding how women’s disadvantages in political and social reality directly relate to under- and misrepresentations in media reality (e.g., Hayes & Lawless, 2015; Hooghe et al., 2015; for an overview cf. van der Pas & Aaldering, 2020). However, such distant explanations of women’s underrepresentation in the mediated reality pay less attention to more proximate gender influences in the mediation process and, thus, underappreciate journalism’s crucial role in mediating societal biases.

**Figure 1.**
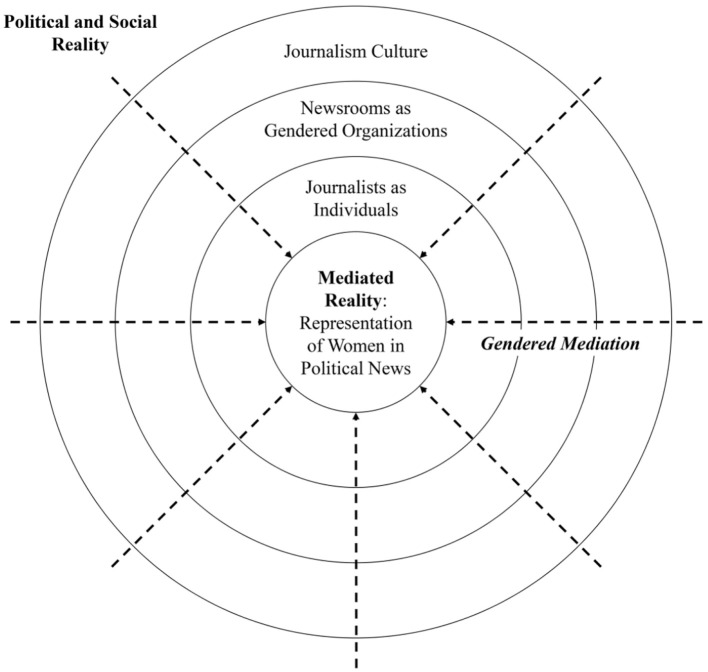
Framework for gatekeeping in the gendered mediation process. *Source.* Own illustration inspired by Gidengil and Everitt (2003); Shoemaker and Reese (2014).

To systematize the role of journalistic factors in the gendered mediation process, we conceptualize the question of women’s representation as a result of journalists’ *gatekeeping* control and approach it from the perspective of the *hierarchy of influences-approach* (Shoemaker & Reese, 2014). This approach considers “factors at multiple levels of analysis that shape media content, the journalistic message system, from the micro to the macro,” and thus at “each level, one can identify the main factors that shape the symbolic reality constituted and produced by journalism” (Reese, 2016, p. 1). In the following, we embed this hierarchy of influences within the gendered mediation process to derive a heuristic framework that systematizes journalistic influences on gender representations in political coverage on three levels: (a) *journalism culture*, (b) *newsrooms as gendered organizations*, and (c) *journalists as individuals.* Figure 1 shows—inspired by the original illustration by Shoemaker and Reese (2014, p. 9)—how these three layers are nested within each other, with journalism culture representing the outermost structural and journalists the innermost individual level. It highlights that the translation of political and social reality into mediated reality requires “passing” all three layers (as indicated by the dotted lines) and that, thus, each layer becomes (potentially) influential for women’s representation within the process of gendered mediation.

### Journalism Culture

Journalism culture has been defined “as a particular set of ideas and practices by which journalists [. . .] legitimate their role in society and render their work meaningful for themselves and others” (Hanitzsch, 2007, p. 369). It encapsulates journalists’ notions of their profession in relation to society as a whole. We therefore locate it at the level of social institutions in the hierarchy of influences-approach, where it expresses the “professional logic of journalism” (Shoemaker & Reese, 2014, p. 103). The level of social institutions “captures the influences attributable to media organizations acting similarly to constitute a larger institution” (Reese, 2019, p. 2), pointing to the fact that journalism’s logic is relational to its political and economic contexts—which then feed back into journalism culture as a collective set of ideas. Accordingly, journalism culture is widely shared among journalists within a geographic and cultural context, with more variance between different contexts than within. One integral part of journalism culture are professional journalistic roles. As “discursive constructions of journalism’s identity and place in society” (Hanitzsch & Vos, 2018, p. 146), these roles express which ideas journalists follow. Thus, while journalism culture expresses journalism’s identity on the institutional level, it becomes manifest—and, therefore, empirically accessible—at the level of individual journalists.

In the past, research on gender in the context of journalism culture has particularly stressed how men and women journalists adapt differently to given cultural norms. Studies initially suggested that men and women may follow different professional roles (e.g., Cassidy, 2008). However, more recent research challenges these assumptions. A large comparative study found that professional journalists’ roles do not differ meaningfully according to their gender (Hanitzsch & Hanusch, 2012, p. 274). Although evidence for gender differences in journalists’ adoption of journalism culture is ambiguous at best, this does not rule out journalism culture as a potential source of gender biases in the actual journalistic outcome. Based on the idea that “‘news professionalism’ has always been defined in male terms” (Lavie & Lehman-Wilzig, 2005, p. 81), we, in contrast, argue that the underlying rationale behind professional roles—and not necessarily gender differences in their adoption—reflects the inherently gendered logic of journalism culture (cf. Vandenberghe et al., 2020). From the plethora of journalistic roles suggested by journalism research (Hanitzsch & Vos, 2018), we focus on three specific roles in which we see the most distinct potential to influence gender representations.

First, the principle of “neutrality,” which is one of the central characteristics of the “neutral” *observer* role (Hanitzsch & Vos, 2018), seems to relate to gendered representations in two ways. On the one hand, early theorizing on gendered “othering” stressed that, in our society, male perspectives are often perceived as synonymous with the norm of the allegedly neutral perspective (Bordo, 1996). Djerf-Pierre (2007) argues that this also applies to journalism and understands “distance” and “neutrality” as an inherently masculine “gender logic in journalism.” Based on the idea that a journalism “that describes reality with dispassionate perspective or ‘distance’” is at the same time one “that consults (male) holders of power as sources,” this logic may also influence gendered representations (Djerf-Pierre, 2007, p. 97). On the other hand, empirical findings based on qualitative research suggest that journalists believing in “neutrality” are particularly prone to overlooking the social-constructivist nature of their work and, thus, at least perpetuate existing gender biases within the political and social reality (Lavie & Lehman-Wilzig, 2005; Vandenberghe et al., 2020). Therefore, we hypothesize:

**Hypothesis 1a (H1a):** Journalists’ identification with a “neutral” *observer* role has a negative influence on the quantitative representation of women in political news coverage.

Second, one central aspect of journalism culture is journalism’s societal responsibility to ensure “provision of access for a diversity of voices” (Hanitzsch & Vos, 2018, p. 149), as it is particularly emphasized by democratic theories. In particular, constructionist and participatory models strive for a popular inclusion of a diversity of voices and, thereby, “would privilege the voices of those who are marginalized in society” (Ferree et al., 2002, p. 307). This undoubtedly applies to women’s voices, given their global marginalization in the political and social reality. If journalists aim to give “the people” a voice in a bottom-up impetus, we can also expect them to be strongly aware of the half of society lacking appropriate representation so far—namely, women. We, therefore, expect that journalists who embrace their role as empowering *access providers* (Hanitzsch & Vos, 2018) are more likely to use women as sources and display them as actors in their news coverage:

**Hypothesis 1b (H1b):** Journalists’ identification with an *access provider* role has a positive influence on the quantitative representation of women in political news coverage.

Third, we assume women’s representation to be connected with the idea of entertainment. Entertainment has early on been argued to be part of journalism culture (Johnstone et al., 1972) because of its ability to generate audience interest and involvement, which has served as a key economic foundation of journalism both historically and in the present (McNair, 2005). In turn, gender-related research has stressed that information is contrasted with entertainment or recreation and that they are constructed as “gendered pairs of opposites in public communication” (Lünenborg, 2006, p. 42, own translation), with entertainment stereotypically seen as connoting the feminine and information as connoting the masculine. More specifically, it has been problematized that journalists may instrumentalize women for entertainment value by depicting them as “eye candy” (Ross, 2007, p. 451) or “sources of visual pleasure” (Jia et al., 2016, p. 1). We, therefore, argue that journalists’ emphasis on entertainment and relaxation is connected to the presence of women in the news. Although the kind of female representation arising from this logic might be questionable, we posit the following:

**Hypothesis 1c (H1c):** Journalists’ identification with an *entertainer* role has a positive influence on the quantitative representation of women in political news coverage.

### Newsrooms as Gendered Organizations

Both journalism research in general (Shoemaker & Reese, 2014) and gender-specific research in particular (Nilsson, 2010) have stressed that news organizations and newsrooms deeply impinge on journalists and journalistic news production. From early on, feminist scholarship has made an effort to debunk the alleged gender neutrality of news organizations and has highlighted that they function instead as “an important location of male dominance” (Acker, 1990, p. 139). While a variety of organizational factors may potentially influence women’s representation, we focus on a specific aspect that has so far received little scholarly attention: the role of gender guidelines in journalistic reporting.

In the last decade, journalism and media federations and nongovernmental organizations, such as the International Federation of Journalists (IFJ, 2009), the United Nations Entity for Gender Equality and the Empowerment of Women (UN Women, 2019), or the European Broadcasting Union (EBU, 2019), as well as individual newsrooms have developed and implemented guidelines for gender sensible reporting and gender-fair language. Such guidelines are insightful, as they can be seen as an expression of collective awareness of gender-related issues; given that the “main explanation for the on-going dominance of White male news sources is lack of awareness” (Vandenberghe et al., 2020, p. 240), this is likely to translate into a higher representation of women in actual news coverage. Despite the potential of media guidelines, knowledge about their effectiveness is scarce and limited to specific topics (e.g., violence against women; cf. Sutherland et al., 2016), with no studies addressing the link between guidelines and the general representation of women.^1^ We therefore test:

**Hypothesis 2 (H2):** Journalists’ consciousness of *gender policies* in their newsroom has a positive influence on the quantitative representation of women in political news coverage.

### Journalists as Individuals

Research inspired by the *hierarchy of influences-approach* has diagnosed “a growing awareness of the supremacy of systemic influences” (Hanitzsch et al., 2010, p. 9) that seem to constrain individual characteristics. Yet, individual factors may be crucial for gender-related aspects of news production. Because of the highly intimate ways in which individuals construct and experience gender (Fiske & Stevens, 1993), journalists default to “doing gender while doing journalism” (Lünenborg, 2006, p. 42). Gender identity is thus woven into journalistic thinking and practices, potentially contributing to a gender-specific selection and depiction of sources and actors (van Zoonen, 1998; Wanta & Craft, 2004). Consequently, Wanta and Craft (2004, p. 124) call women editors in news organizations “relatively powerful gatekeepers”.

Indeed, past content analyses of media coverage routinely linked journalists’ own gender to the gender of featured actors, with male journalists representing male actors to a significantly stronger extent (Armstrong, 2004; De Swert & Hooghe, 2010; Leiva & Kimber, 2020; McMillin & Weaver, 1996; Rodgers & Thorson, 2003; Zoch & Turk, 1998). This relationship has also been shown in the case of women editors (Shor et al., 2015) and in the fictional genres of media production (Linke & Prommer, 2021). Reasons for journalists’ gender homophily include gender-specific socialization (Rodgers & Thorson, 2003), gendered networks, routinely used contact lists (Hooghe et al., 2015), and slanted distributions of women and men across journalistic beats and topics (Craft & Wanta, 2004; De Swert & Hooghe, 2010; Sjøvaag & Pedersen, 2018).

However, other studies did not replicate the finding of journalists’ own gender as a gatekeeping influence on gender representations or found only minimal effects (Cann & Mohr, 2001; Liebler & Smith, 1997; Piper-Aiken, 1999). The notion of a “gender determination hypothesis” has also been criticized as too simplistic, given that journalists lack the necessary freedom to adjust their performance according to individual parameters (Hanitzsch & Hanusch, 2012, p. 258). In light of the inconclusive evidence, we plead for a more rigorous test with a research design that controls for both professional and organizational factors as well as parameters of the actual news outcome. Expanding on existing content-analytic research, we suggest the following:

**Hypothesis 3 (H3):** Journalists’ male *gender* has a negative influence on the quantitative representation of women in political news coverage, whereas journalists’ female gender has a positive influence.

So far, we have discussed which aspects of journalistic news production can be expected to influence gender representations. In addition to knowing *where* gender influences originate from, it is important to understand *how* these influences become efficacious in the process of news production. To capture the complexity of journalistic decision-making and possibly uncover additional sources of potential gender bias, we thus complement our set of hypotheses with an explorative research question:

**Research Question 1:** How do journalists negotiate their socialization within a professional culture, organizational forces, and their own gender when it comes to representing women in political news coverage?

## Mixed-Methods Study Design

To test our deductively derived hypotheses as well as to answer our explorative research question, we realized a mixed-methods research design consisting of three sequential nested data collection phases: (a) a manual quantitative content analysis of Austrian political news coverage, (b) a quantitative survey among the authors of the analyzed news items, and (c) qualitative reconstructive interviews with a selection of these authors.

### Austria as a Case

As an object of study, Austria is comparable to other European countries, but it has distinctive features relevant in the context of gendered representations. Belonging to the Democratic Corporatist Model (Hallin & Mancini, 2004), it is characterized by a fairly pronounced (and still ongoing) professionalization of journalism, a (still) high circulation of printed newspapers, and a strong (though in recent years steadily decreasing) role of public broadcasting. Austria exhibits a high level of media concentration with a powerful tabloid newspaper (“Kronen Zeitung”) still holding around a quarter of the newspaper market and shows a high circulation of tabloid-like free daily newspapers (Grünangerl et al., 2021; Lohmann & Riedl, 2019). Concerning gendered representations, however, Austrian tabloids—at least in quantitative terms—offer a higher degree of women’s representation than many other outlets (Hayek & Rußmann, 2020; Seethaler, 2015). Still, women only account for 14% of featured actors in the general news coverage (Magin & Stark, 2010), are included in 18% of news items (Seethaler, 2015), and remain consistently underrepresented in coverage of electoral campaigns (Hayek & Rußmann, 2020; Melischek & Seethaler, 2017).

Journalism culture in Austria corresponds to a typical “Western” culture insofar as it places high importance on critical but detached (political) information and a wide rejection of interventionism (Hanitzsch, Hanusch, et al., 2019; Hanitzsch, Seethaler, & Wyss, 2019; Riedl, 2019). We thus expect the *observer* role to be the most and the *entertainer* role to be the least supported. Due to Austria’s small size, Austrian journalists report close and intense interactions with stakeholders (Maurer & Riedl, 2020), highlighting the role of personal connections. The share of female journalists has increased from 42% in 2006 to 47% in 2018/19; nevertheless, women journalists are still considerably underrepresented in leading positions. Austrian journalists’ awareness of existing gender guidelines at their organization has increased substantially, rising from 20% in 2008 to 35% in 2018/2019 (Kaltenbrunner et al., 2020). Although the conditions of journalistic news production and its outcomes are rather well documented from a gender perspective in Austria (Kirchhoff & Prandner, 2016; Thiele, 2019), they have hardly been empirically connected.

### Quantitative Content Analysis

To capture women’s representation in political news coverage, we performed a quantitative manual content analysis of 3,539 political news items with national political relevance published in 2018,^2^ which was part of a larger research project. News items were determined to be politically relevant with the help of a search string,^3^ which was applied to a commonly used database. To ensure a broad and diverse sample, 12 news outlets were analyzed, which were selected according to their reach within the sectors of broadsheet newspapers, tabloid newspapers (both offline and online), regional newspapers, public service TV, public service radio, and public service online news.^4^ Coding was executed by eight graduate students who were trained in weekly sessions. Intercoder reliability was evaluated by Brennan and Prediger’s (1981) Kappa, which is particularly appropriate for skewed distributions. It was calculated based on randomly selected news items from each outlet (*n* = 135).

For each news item, up to four main actors could be coded. We defined an *actor* as a person (e.g., “ordinary” citizen, political representative, expert) or collective (e.g., party) who (in)directly expresses an opinion and/or shares an assessment. This narrow definition of actors has the advantage of capturing only actors with a reasonable degree of societal representation and participation in public discourses. Reliability was tested according to a verbatim coding of an actor’s actual name or label (Brennan & Prediger’s κ = .67), regardless of the position within a news item. Actor gender was coded as female, male, or as a third, nonbinary gender based on names and ascribed pronouns (κ = .95) for individual actors. The third option was coded in three cases of the whole content analysis data set and only remained within the data set with respective survey data in two cases and, therefore, could not be considered. Because of the research interest asking for the role of gender, collectives that were coded as actors were also not considered in the following analyses.

We use the data from actor coding to construct three measures of women’s representation as our dependent variables. We focus on quantitative representation because media visibility feeds gendered (mis)perceptions about social roles with “significant consequences for social stratification” (Shor et al., 2015, p. 960). The first and most rudimentary measure is a dummy variable indicating the *presence* (= 1) of at least one female actor in a news item. This measure reflects the minimal bar for women’s representation and is the least conservative indicator of journalists’ gender-balanced reporting. A second measure more precisely quantifies women’s representation as the *number* of female actors (0–4) within a news item. This measure adds information about women’s absolute representation and circumvents the practice of including a single woman as a token. Third, we calculate the *gender ratio* of female (*n_f_*) to male (*n_m_*) actors within a news item as (*n_f_* − *n_m_*) / (*n_f_* + *n_m_*). Negative values therefore indicate a relative underrepresentation of women in a given item. This final measure presents the most robust indicator of women’s representation because it is relative to the representation of men. To control for the thematic context of gender representations, we additionally attributed whether the coded topic of the news item (Brennan & Prediger’s κ = .66) is stereotypically *feminine*. For this variable, we recoded a list of 31 topics as stereotypically *feminine* (= 1) or *not* (= 0) based on existing literature (e.g., Meeks, 2012).^5^

### Quantitative Survey

In a second step, we invited all authors of coded news items whose contact details could be researched to participate in a quantitative online survey. Between September and December 2019, 49% of the contacted journalists completed the survey, which yielded a sample of 208 journalists authoring 789 news items. Content analysis and survey data were matched at the level of the individual news item and individual journalist. To gain more robust findings, we only included journalists who authored at least three news items (e.g., as done by Raemy et al., 2018). This resulted in a final sample of 108 journalists responsible for 654 news items.

The survey contained our main independent variables. First, journalists’ identification with specific journalistic roles was measured with three commonly used items (wording: Hanitzsch, Hanusch, et al., 2019; Hanitzsch, Seethaler, & Wyss, 2019). The three items asked for the extent to which journalists agreed on a 5-point scale (from 1 = “not at all” to 5 = “strongly agree”) with the following statements: “my [their] job is all about [. . .]” (a) “being a detached observer” (*observer* role), (b) “letting people express their views” (*access provider* role), and (c) “providing entertainment and relaxation” (*entertainer* role). We additionally measured journalists’ perceived *autonomy* with 2 items on a 5-point scale, which were then averaged into one control variable (“How much freedom do you have in deciding which aspects of a story should be emphasized?” and “[. . .] selecting news stories you work on?”) (cf. Hanitzsch, Hanusch, et al., 2019). To measure journalists’ consciousness of *gender policies*, respondents were asked whether guidelines (“written rules for gender-sensible presentation/language”) exist in their newsrooms as also used in former studies in Austria (Kaltenbrunner et al., 2020). In terms of journalists’ *gender*, they could indicate to identify as female, male, or third, nonbinary gender. None of the respondents chose the third option.

### Qualitative News making Reconstruction

In a third step, from September to November 2020, we conducted qualitative interviews with 24 selected journalists (12 men and 12 women), who were equally distributed within the three sectors of (a) broadsheet newspapers, (b) tabloid newspapers (both offline and online), and (c) public service media (TV and radio). We used the method of “Newsmaking Reconstructions” (Reich & Barnoy, 2020) in a qualitative way, in which journalists deconstruct and reconstruct the processes of news making, thus illuminating the “story behind the story” (Brüggemann, 2013). By discursively depicting one’s own work process step by step, its “logic and reasoning” becomes tangible (Reich & Barnoy, 2020, p. 973). This approach provides an in-depth understanding of how journalists negotiate their own intentions and perceptions within the news production process, and it has recently been successfully applied in gender-related research (Vandenberghe et al., 2020).

In each interview (during 61 min on average), two to three news items were reconstructed. The news items were either from the quantitative content analysis or, to ensure that journalists were able to remember at least some sufficiently, were chosen from journalists’ newer work (i.e., work published in the weeks before the interviews). The interviews were part of a larger project on normative media performance in general. Within each reconstruction, journalists were initially asked to openly recapitulate each news item’s genesis, detecting their own relevance structures; male or female sources could appear as impulses for news coverage here. Journalists were then invited to consider whether there were specific actors to whom they were determined to give a voice and whether actors were relevant in the research process but were not included in the final “story.” Only if gender did not appear so far were journalists asked to reflect on how the sources’ gender (may have) played a role. In this whole process, more in-depth follow-up questions aimed to ensure that the descriptions of news decisions were tied back to the organizational context of the newsroom.

The three data sources were integrated into one MAXQDA data set to allow for a comprehensive analysis. The interview material was reviewed and, in a first step (Nawratil & Schönhagen, 2021), inductively examined for gender-relevant passages. In a second step, and with regard to compatibility with the quantitative data, the identified patterns of reasoning and negotiation were connected to the levels of journalism cultures, organizations, or individuals, uncovering the complexity of gendered news decisions. In the next section, we draw from the combined data set to discuss our hypotheses (**H1–H3**) while contextualizing and enriching them with journalists’ own experiences (**RQ1**).

## Findings

### Women’s Underrepresentation in Political News Coverage

To substantiate the claim of women’s underrepresentation in Austrian news coverage, we first provide a descriptive account of all three representation measures in both the full content analysis data and the merged data set with matched journalists (see Table 1). Across measures, women actors are, in fact, underrepresented. In all, only 25% of the news items include at least one woman’s viewpoint. While this is a somewhat higher share than found in past studies (e.g., Seethaler, 2015), it remains widely below the representation of men, whose perspectives are covered in 68% of all articles. Similarly, news items contain only 0.29 women actors on average but 1.15 men actors, with a heavily male-skewed gender ratio of −0.603.^6^

**Table 1.** Women’s Representation in Political News Coverage in Austria.

	Presence (% of news items)		Number (within average news item)		Gender ratio^a^
Data basis	Women	Men	Women	Men	Women vs. Men
All news items (*n* = 3,509)^b^	25.1	68.2	0.29	1.15	−0.603
All news items with respective survey data (*n* = 654)	28.1	71.7	0.34	1.23	−0.583
News items by men journalists (*n* = 199)	24.0	72.2	0.29	1.16	−0.637
News items by women journalists (*n* = 413)	38.2	69.3	0.45	1.33	−0.446
χ^2^ / *t*	12.61	0.39	2.95	1.68	2.86
*p*	<.001	.533	.03	.351	.005

*Note.* χ^2^ tests were used for the association of journalists’ gender with the presence of at least one woman or man actor. Student’s *t* tests were used for differences in the average number of women actors, men actors, and their ratio according to journalist gender; none of the χ^2^ tests between the full and merged data sets were significant.

a Range: −1 (*only men*) via 0 (*balance*) to 1 (*only women*). ^b^Data are weighted according to the ratio between population and sample size for each media outlet.

To understand which journalistic factors systematically influence women’s representation, we ran separate generalized linear mixed models (GLMM) by using the R-package *mgcv* (Wood, 2017) for all representation measures (see Table 2), which serve as the basis for the remainder of the results section. The models acknowledge the multilevel structure of our data, with three or more news items nested within journalists authoring them.

**Table 2.** Predictors for Women’s Representation.

Generalized linear mixed model (Logit/Gaussian)	Presence of women		Number of women		Gender ratio	
Predictors	Odds ratio	*p*	Estimate	*p*	Estimate	*p*
(Intercept: Journalists)	0.19	.272	0.12	.736	−1.06	.016
Journalism Culture						
Observer role	0.97 (0.14)	.842	−0.01 (0.03)	.669	0.05 (0.04)	.211
Access provider role	1.19 (0.12)	.163	0.06 (0.03)	.045	0.02 (0.04)	.619
Entertainer role	1.31 (0.11)	.015	0.05 (0.04)	.056	0.09 (0.03)	.013
(Control: perceived autonomy)	1.30 (0.25)	.290	−0.06 (0.06)	.309	0.11 (0.08)	.165
Gendered Organizations						
Consciousness of gender policies	0.85 (0.41)	.699	−0.06 (0.10)	.560	−0.07 (0.13)	.608
(Control: tabloid media outlet)	0.80 (0.31)	.462	−0.07 (0.07)	.321	−0.00 (0.09)	.978
Journalists as Individuals						
Male gender	0.53 (0.25)	.010	−0.14 (0.06)	.025	−0.24 (0.08)	.002
(Control: stereotypically feminine topic)	0.85 (0.26)	.494	−0.02 (0.07)	.766	−0.01 (0.08)	.966
Education	1.12 (0.12)	.349	0.04 (0.03)	.185	0.04 (0.04)	.236
Age	0.98 (0.01)	.179	0.01 (0.01)	.251	0.01 (0.01)	.432
*n* (news items) / *R*^2^ Nagelkerke / *AIC*	536 / .092 / 628.02		536 / .070 / 987.09		427 / .124 / 879.04	

*Note.* Numbers in parentheses are standard errors. AIC = Akaike Information Criterion.

### Journalistic Roles as a Source of Gendered Representations

At the outermost layer of our framework, we placed journalistic roles as part of journalism culture. As assumed, the surveyed journalists strongly and most concordantly supported the *observer* role (84% “rather” or “strongly agree”), while an *access provider* and *entertainer* role were less endorsed (46% and 26%).^7^ These roles were used as predictors to test hypotheses **H1a–c**^8^ (see Table 2). Holding organizational and individual factors constant, journalists who self-identify more strongly with an *access provider* role significantly include more women in a news item; however, they are not more likely to include at least one woman in their reporting or provide a more equitable gender ratio per news item. Journalists’ identification with an *entertainer* role has a significant positive influence on the presence of women as well as on the gender ratio but not on the number of women; journalists oriented toward entertainment seem to care for a literal “token woman” but do not necessarily include more women in absolute terms per news item. In all cases, however, these effects are relatively small. In contrast to our expectation, an *observer* role shows no association with gender representations. We therefore reject **H1a** but find support for **H1b** and **H1c**, though only for some measures.

Regarding our explorative research question, several journalists have referred to their professional identity in the reconstructive interviews. They invoke their role as “neutral” *observers* when constructing women’s underrepresentation as an issue that is mainly driven by external factors located in the gendered social reality: “The simple fact is that in management boards, in supervisory boards, in political functions, you still have a male dominance” (J5). As a commitment to journalistic neutrality, journalists see themselves limited to mirroring the dearth of powerful women in societal institutions: “The health minister is a man [. . .], I can’t pull a female health minister out of a hat” (J4). However, in contrast with this perceived lacking availability of female sources, some journalists also commend the increasing awareness among organizations of the need to display women’s voices at the forefront of their public communication. In this case, neutral observation has less to do with the faithful depiction of the “true” social reality and more to do with the practical and situational accessibility of women experts. These contradictory lines of argument may also cause the lack of evidence in the quantitative models.

Journalists’ references to the *access provider* role align with an explicit intention to include women more systematically and, unlike the relatively passive *observer* role, underscore journalists’ agency. Rather than accepting men’s overrepresentation as a default, they dismiss the argument of reduced accessibility as “a stupid excuse [. . .] because there are very smart women on every topic who can be given a chance to speak” (J20). In line with the theoretical argument, journalists want to give voice to *the* people as a whole and not only to a specific group: “Journalism must somehow depict the whole picture—and that includes women” (J4). Crucially, deliberately providing access to women also entails recognizing journalists’ potential to (re-)shape gender relations: “If people are confronted with only men as [. . .] explainers, then of course that also cements gender relations” (J21). Despite this intention to include women, journalists report specific gender-related barriers, such as women’s perceived lack of confidence (“the problem is that women never dare to say anything,” J23)—as it represents a form of “gendered interactions” (Nilsson, 2010, p. 3)—or care work obligations (“very often [. . .] women cannot set up childcare so quickly,” J4).

Journalists invoke the professional role of the *entertainer* when discussing—at least assumed—audience expectations. On the one hand, journalists report including women not because they substantively contribute to the news story but because they are thought to be valuable from an entertainment perspective. For example, women may fit well “atmospherically” (J5), illustrate the “very lively” (J6) tone of an event, or add emotive value when they are featured as celebrities. Working with entertainment can also be a strategy to cater specifically to journalists’ perceived interests among their female audience: “We know that female readers also like to read the voices of women” (J8). On the other hand, men’s representation, too, can be associated with entertainment because their quotes are seen as potentially more newsworthy. Again reflecting gender-stereotypical assumptions, a journalist almost resignedly states that “the persons [. . .] who dare to speak more pointedly, are, as a general rule, men. Women are more cautious, reserved, reflected and therefore simply more boring in their expression” (J6).

### Limited Effect of Gender Guidelines

In the second layer of newsrooms as gendered organizations, we investigated the effect of journalists’ consciousness of gender guidelines on women’s representation^9^; only a minority of 12% of the surveyed journalists were aware of such guidelines. In the regression models (see Table 2), we find no significant influence of these policies on any of the representation measures. We therefore reject **H2**. At least at first glance, this finding casts doubt on the effectiveness of gender guidelines. The qualitative analysis suggests that this may be the case because journalists interpret gender-related measures mainly within the news production process itself (e.g., “it is always examined [. . .] how many men and women write on the comment page,” J21), but they remain rather disconnected from the actual content of their journalistic output.

Paradoxically, increased awareness—possibly raised through such guidelines—may also have an adverse effect by creating the impression of a false safeguard against gender-insensitive reporting. For example, one journalist even states that the equal representation of women may be “already partially taken for granted, so people no longer give much thought to ensuring that women also have their say” (J22). Without altogether dismissing the potential of gender guidelines as an organizational tool to increase diversity, these arguments could at least partially explain why a perceived influence of gender guidelines does not necessarily translate into a higher representation of women in actual news coverage.

### Journalist’s Own Gender as Determinant

In the third layer, that of individual journalists, we tested the influence of journalists’ own gender^10^ (see Table 2). We find a consistent and negative effect of journalist gender on all representation measures, and this is the strongest predictor in all models. Even when controlling for professional, organizational, and other individual factors, male journalists significantly underrepresent women actors in their reporting. Fully supporting H3, male journalists (a) are half as likely to include at least one woman actor in a news item, (b) cover fewer women in general, and (c) have stronger gender imbalances within news items than female journalists. In descriptive numbers (see Table 1), only 24% of male journalists’ news items contained at least one woman as a central actor, with an average number of 0.29, whereas news items authored by female journalists had 38% and an average of 0.45. Interestingly, the bivariate comparisons reveal that journalists’ gender only affects women’s representation, whereas men actors are equally covered by both men and women journalists.

Despite its strong effect in the quantitative models, journalists hardly addressed their gender in the reconstructive interviews. An exception is a middle-aged woman with a migrant background who mentions the formative influence of gender and other attributes of identity: “My biography and my life are different from that of the average Austrian [male] journalist, especially of the older generation [. . .]. My approach and my reality are different” (J2). In this idea, the qualitative analysis points to an intersectional connection between gender, age, status, and ethnicity but gives us limited insight into the role of journalists’ gender as an isolated construct. While social desirability may, of course, account for some of the absence of gender in the interviews, another plausible explanation is that gender acts as a psychological (and, to some extent, methodological) “blind spot.” Because gender marks a seemingly natural and highly integrated part of the personal identity, journalists may not be able to assess to what extent its structuring influence extends to their social and professional lives. In particular, male journalists may, for example, seem to “naturally” build a more male-dominated network of sources and may not recognize the role of their own gender in doing so.

## Discussion and Conclusion

The aim of this article is to shed light on how journalism mediates the structural gender dynamics of societal and political reality in terms of women’s representation in political news coverage. We first derive a multilayered framework of gendered influences on journalists within journalistic news production, originating from the level of journalism culture, gendered organizations, and journalists as individuals. Combining three data sources (content analysis, journalist survey, and reconstructive interviews), we empirically link these journalistic factors to women’s underrepresentation in political media coverage. In our quantitative analysis, journalists’ gender emerges as the strongest journalistic driver of the amount of political coverage dedicated to the voices and perspectives of women. Furthermore, we find that specific professional roles—but not journalists’ consciousness of gender guidelines at the newsroom level—affect gender representations, though with rather weak effects. The results from the qualitative and quantitative analyses are mostly congruent and in line with related qualitative research (e.g., Vandenberghe et al., 2020).

One of our most striking findings is the crucial role of journalists’ own gender in whether and to what extent women appear in political news, with a stronger tendency for male journalists to underrepresent women. Although there are several arguments as to why a journalist’s gender matters, its strong influence on gender representations stands in contrast to more general findings in journalism studies, which show that the influence of journalists’ individual parameters is limited by structural parameters. Our findings challenge this assumption, suggesting that gender may be “special” in comparison to other individual categories (Fiske & Stevens, 1993). Yet, the strong effect of gender also implies a considerable degree of agency in journalists’ actions when it comes to representing women.

However, our account of gender also raises conceptual and methodological questions. There is some conceptual unclarity what the observed effect of gender “really” means. On the one hand, it does not imply that there is an inherently “feminine” or “masculine” way of doing journalism. On the other hand, it still remains an open question to what extent our findings of “individual gender identity [. . .] are also products of organizational processes and pressures” (Acker, 1990, p. 140), which were not controlled for. Thus, future research on newsrooms may focus on still “hidden” organizational and/or professional factors (e.g., gendered interactions or the gendered assignment of specific stories). For instance, such “gendered interactions” (Nilsson, 2010, p. 3) could reflect a society’s more general gender-related values, which, as social beings, journalists hold beyond their professional views. As Austria is comparably conservative in terms of gender equality (European Values Study [EVS], 2020), the effect of journalists’ genders might be less pronounced in more progressive societies.

With regard to professional roles, our study expands upon existing qualitative research on journalistic roles and gender (e.g., Vandenberghe et al., 2020) by providing the first quantitative evidence of the embeddedness of gendered news decisions in the larger framework of journalism culture and by opening up additional theoretical directions. Our qualitative analysis draws particular attention to the multidimensional character of professional roles. For example, the idea of mirroring “reality” might work against women because journalists’ position as allegedly “neutral” observers perpetuates existing societal biases. Yet, this also implies that the continued thrusting of women into positions of power and the efforts of organizations to more actively put women on center stage could positively affect gender representations in the long run. Thus, our study emphasizes the theoretical value of journalism culture for the study of gender representations while also underlining the complexity of its empirical application.

Against our expectation, this study did not find any evidence that guidelines for gender-sensitive reporting affect the actual representation of women. This finding needs to be understood within the context of only a minority of surveyed Austrian journalists being aware of such guidelines. Thus, it is possible that guidelines may also remain ineffective at present because we have not yet achieved the necessity of a “critical mass” of journalists within a newsroom actively advocating for gender equality (e.g., Craft & Wanta, 2004; Eckert & Assmann, 2021). However, there is also a need for conceptual clarification of what constitutes gender guidelines, as they can be a code of conduct, a loose set of recommendations, or a fully integrated part of newsrooms’ organizational structure. Therefore, future studies should collect more fine-grained information about the specific contents of guidelines through content analyses and compare journalistic output from a larger selection of organizations—both with and without guidelines. Although the consciousness of gender guidelines does not suffice to ensure adequate representation of women in journalistic output, our findings highlight the importance of measures that directly translate awareness into actual practices by tackling the issue of limited resources in the process of news production. Examples of such tools could be databases of women experts on the level of newsrooms, such as those recently implemented by the Austrian Public Broadcasting Corporation ORF (Rössner & Weissenböck, 2021).

This investigation has limitations. First, our quantitative models only explain a limited amount of variance in women’s underrepresentation. Although this can be partially traced to the considerable time span between the content analysis and the survey data, it raises the question of rather stable determinants versus situational influences on gendered representation within the news production process. Second, as a first attempt to empirically weigh journalistic factors against each other on different levels, we restricted our analysis to the quantitative representation of women. Given the importance of qualitative aspects of media representation, future studies are invited to additionally explore how multilayered journalistic factors in news production influence the different forms, depictions, and types of coverage. Although it may be true that journalists cannot simply pull women out of a hat, shedding light on the role of journalistic factors in the gendered mediation process can make the normative goal of gender-balanced media coverage seem less magical.

## Notes

1. An exception is a study by De Swert and Hooghe (2010), who found a slightly *lower* share of women in the news produced by a broadcaster with a new gender equality policy. However, the study only compared two competitors in a specific context.

2. 26.5.−15.06. + 29.06.−05.07.2018, 17.09.−07.10. + 22.10.−28.10.2018

3. ÖVP OR FPÖ OR SPÖ OR NEOS OR „Liste Pilz“ OR Nationalrat* OR Kultusgemeinde OR Caritas OR Diakonie OR (( Grünen“ OR „der Grünen“ OR „den Grünen“ OR Bundesrat* OR *kanzler* OR *präsident* OR *minister* OR *regierung* OR *parlament* OR *klubob* OR *gerichtshof* OR *partei* OR Landtag* OR Landeshaupt* OR Landesrat* OR *senat* OR Stadtrat* OR Gemeinderat* OR Magistrat* OR Frontex OR Europol OR EZB OR Zentralbank OR Europarat* OR europäisch* OR UNO OR „UN-“ OR OSZE OR G7 OR G20 OR OECD OR NATO OR Terror* OR Botschaft* OR Diplomat* OR Opposition* OR *abgeordnete* OR Untersuchungsausschu* OR Kirche OR Islam* OR jüdisch* OR *kammer* OR Gewerkschaft* OR Industriellenvereinigung OR Nationalbank OR *bürgerinitiative* OR *abstimmung* OR Volksbegehren* OR *demonstra* OR Amnesty OR Greenpeace OR „Global 2000“ OR „Ärzte ohne Grenzen“ OR antifaschis* OR identitär* OR faschis*) AND österr*) OR (( OR *gerichtshof* OR *parlament*) AND („EU“ OR „EU-“) AND österr*).

4. Die Presse, Der Standard, Kronen Zeitung, Kleine Zeitung, ZIB 20, Zeit im Bild, ZIB 2, Ö3 Frühjournal, Ö3 Journal um fünf, derstandard.at, krone.at, orf.at

5. Stereotypically feminine topics were policies related to health care, humanitarian activity, pension, family, poverty, equality (including gender equality), culture, education, and religion; other topics were the constitution of the political system, political parties, politicians as persons, politicians as private individuals, political culture, domestic security, and policies related to foreign affairs, military, the EU, economy, transport and infrastructure, energy, tax, finance and fiscals, labor market and employment, migration, urban and regional planning, sports, law, media, science, agriculture, and environment, as well as other topics.

6. Note that the numbers within the merged subsample of the content analysis, for which survey data are available, vary slightly but not significantly.

7. Note that, due to the mixed-methods sampling approach, which should result in the best possible representativeness of the content analysis data, the descriptive results of the survey data can only claim a lower representativeness than other recent surveys among journalists in Austria (Hanitzsch, Seethaler, & Wyss, 2019; Kaltenbrunner et al., 2020).

8. We control for journalists’ perceived autonomy to reflect the argument that journalists may not have enough autonomy to decide independently who they cover.

9. Given that tabloid news outlets offered a widely higher share of articles with female actors in past content analyses, we used tabloid news outlet as a dummy control variable here.

10. Controlling for the stereotypicality of the news item topics allows us to isolate the effect of journalists’ gender from the uneven distribution of interests, topics, and beats among actors and journalists (e.g., De Swert & Hooghe, 2010; Vu et al., 2018).
